# Supplementary material for: The Quality Evaluation of Avocado Fruits (Persea americana Mill.) of Hass Produced in Different Localities on the Island of Tenerife, Spain
Source: Foods. 2024 Mar 29;13(7):1058. doi: 10.3390/foods13071058 (PMC11011534; doi:10.3390/foods13071058)
Supplement: Supplementary file 1 [file foods-13-01058-s001.zip › foods-2917917-supplementary.pdf]

Supplemetary Table S1. Analysis of irrigation water at different locations.

Location	pH	EC (mS cm^{-1})	CO_3^{2-} (meq L^{-1})	HCO_3^- (meq L^{-1})	SO_4^{2-} (meq L^{-1})	Cl^- (meq L^{-1})	NO_3^- (mg L^{-1})	Ca^{2+} (meq L^{-1})	Mg^{2+} (meq L^{-1})	K^+ (meq L^{-1})	Na^+ (meq L^{-1})
BU	9.3	0.91	0.57	6.53	0.95	1.61	n.d.	0.37	3.10	0.64	5.2
SI	8.2	0.81	n.d.	6.38	n.d.	1.62	n.d.	0.37	2.90	0.63	4.10
OR	7.0	0.31	n.d.	0.95	n.d.	1.58	18	0.43	0.62	1.61	0.25
GU	8.9	1.06	1.23	3.21	1.65	3.74	28	0.89	2.05	0.37	6.4
RE	7.3	0.70	n.d.	2.00	2.22	2.25	69	0.65	1.30	0.61	4.5
SA	7.7	1.20	n.d.	7.60	0.54	2.23	n.d.	0.98	1.83	0.81	8.5
TA	9.3	1.17	1.33	6.59	0.36	1.80	n.d.	0.37	3.91	0.64	7.0

n.d. (not detected). EC (electrical conductivity).
